# Unresolved Complexity in the Gene Regulatory Network Underlying EMT

**DOI:** 10.3389/fonc.2020.00554

**Published:** 2020-05-12

**Authors:** Deborah P. Lavin, Vijay K. Tiwari

**Affiliations:** The Wellcome-Wolfson Institute for Experimental Medicine, School of Medicine, Dentistry and Biomedical Science, Queen's University Belfast, Belfast, United Kingdom

**Keywords:** EMT, cancer, gene regulation, epigenetics, signaling, transcription factors, chromatin

## Abstract

Epithelial to mesenchymal transition (EMT) is the process whereby a polarized epithelial cell ceases to maintain cell-cell contacts, loses expression of characteristic epithelial cell markers, and acquires mesenchymal cell markers and properties such as motility, contractile ability, and invasiveness. A complex process that occurs during development and many disease states, EMT involves a plethora of transcription factors (TFs) and signaling pathways. Whilst great advances have been made in both our understanding of the progressive cell-fate changes during EMT and the gene regulatory networks that drive this process, there are still gaps in our knowledge. Epigenetic modifications are dynamic, chromatin modifying enzymes are vast and varied, transcription factors are pleiotropic, and signaling pathways are multifaceted and rarely act alone. Therefore, it is of great importance that we decipher and understand each intricate step of the process and how these players at different levels crosstalk with each other to successfully orchestrate EMT. A delicate balance and fine-tuned cooperation of gene regulatory mechanisms is required for EMT to occur successfully, and until we resolve the unknowns in this network, we cannot hope to develop effective therapies against diseases that involve aberrant EMT such as cancer. In this review, we focus on data that challenge these unknown entities underlying EMT, starting with EMT stimuli followed by intracellular signaling through to epigenetic mechanisms and chromatin remodeling.

## Introduction

Epithelial to mesenchymal transition (EMT) is a dynamic reversible process and a fundamental mechanism occurring during embryogenesis and organ development, wound healing and fibrosis, and cancer (1–3). The reverse process (MET; mesenchymal-epithelial transition) is vital for secondary tumor formation and also occurs during embryogenesis with multiple rounds of EMT and MET required for correct formation of complex organs and specialized cellular structures (4, 5). Therefore, understanding EMT is not only beneficial in terms of developing novel cancer therapies, but enables us to fully elucidate the mechanisms behind wound healing and organ fibrosis—processes implicated in diabetic complications (6, 7), heart disease (8), and immunocompromised conditions like cystic fibrosis (9).

Gene regulation controls whether a gene is expressed or silenced, and encompasses transcription factors, epigenetic modifications, chromatin remodeling, higher order chromatin structures (such as looping), and non-coding RNAs e.g. microRNAs (miRNAs) and long non-coding RNAs (lncRNAs). In EMT, transcription factors such as Snail, Zeb1/Zeb2, and Twist, bind to specific promoter and enhancer DNA sequences and work in unison with epigenetic regulators (such as G9a and LSD1) and chromatin remodeling machinery to drive transcription of pro-mesenchymal genes and prevent epithelial gene transcription (3, 10). The chromatin remodelers determine how accessible these DNA sequences are (11); therefore, they determine gene expression during both developmental and disease-related EMT. Like EMT, epigenetic modifications—such as DNA or histone methylation, and histone acetylation—are also reversible, and have been shown to play a pivotal role in EMT regulation, which makes them attractive targets for novel chemotherapeutics (12). Interactions between key EMT transcription factors and enzymes that modify DNA/histones have been reviewed in detail (3, 10, 13, 14).

Nucleosomes (histone octamers) are the basic packaging unit of DNA—the “beads on a string”—which confer 5- to 10-fold compaction of the genome and form arrays that fold hierarchically into higher-order (50-fold and higher) chromatin structures (15). These structures, which include multiple chromatin loops, can repress gene transcription in both stem cells and adult cancer cells, for example the TF GATA4 (16). In addition, PcG proteins and repressive chromatin methylation marks work together to preserve these loops (16). The role of chromatin looping in EMT however is unknown.

Non-coding RNAs represent another layer of epigenetic regulation owing to the susceptibility of their promoters to epigenetic modifications (10, 17). Much is known about the role of non-coding RNAs, such as the miRNA-200s family of microRNAs, in EMT (10, 18–20), with recently published data also showing the involvement of microRNAs [miRNA-151a (21)] and long non-coding RNAs [MYOSLID (22)] in partial EMT. Conversely, the long non-coding RNA NKILA which is upregulated by TGFβ, suppressed TGFβ-induced EMT and tumor metastasis *in vivo* through negative regulation of downstream TGFβ signaling (23). All of these elements add another layer of complexity and culminate in highly intricate gene regulation.

Unknowns in each area of gene regulation in EMT compromise not only our fundamental understanding of these mechanisms but interfere with our knowledge of EMT pathogenesis. Without this information, we cannot develop critically needed cancer therapeutics targeting EMT, as in addition to driving tumorigenesis and metastasis, EMT confers chemoresistance and helps tumor cells evade destruction by the immune system (24). Advances in the field of regenerative medicine (i.e., cellular reprogramming to restore organ functionality) also rely on deciphering these unknowns. Chemotherapeutics that inhibit DNA methylation (e.g., 5–aza-2′-deoxycytidine, Guadeticabine), histone deacetylation (e.g., Vorinostat, Mocetinostat), and interfere with recognition of acetylated lysine residues (e.g., BRD4 inhibitors such as JQ1, MS417), are promising as they, respectively, restore epithelial phenotypes/reactivate tumor suppressor proteins (10, 13, 25), reduce growth/antagonize Zeb1-mediated miRNA-203 repression (10, 26), and suppress the MYC TF, invasion, and tumorigenicity (10, 25). While these targeted therapies may have a synergistic effect with platinum-based chemotherapies and may sensitize cancer cells to therapies that induce DNA damage (26), these inhibitors are not perfect due to potentially adverse activation of otherwise latent genes, and their somewhat limited effect on solid tumors (10). Here, we discuss unknown epigenetic entities in the gene regulatory network underlying EMT.

## Thinking Outside the Cell—Novel EMT Stimuli

**Figure 1:**
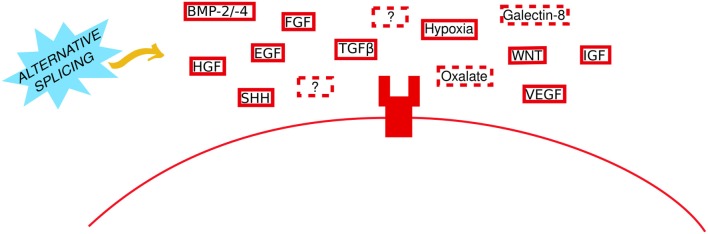
Thinking outside the cell in EMT. Established and novel extracellular stimuli bind to their particular receptors to initiate EMT. A generic transmembrane receptor is shown here for illustrative purposes. Solid and dashed red boxes represent established and novel stimuli, respectively. Question marks represent unknown stimuli. The yellow arrow indicates that the role of alternative splicing (sky blue spiked shape) remains to be investigated.

Extracellular stimuli are the initiating factors that drive signaling and cellular effects and are often the first point of regulation in disease; pharmacological antagonism of deleterious stimuli or their receptors is often the first treatment option or the only option if the mechanism through which the stimulus mediates its effects are unknown. For example, administration of anti-VEGF is routinely performed in diabetic retinopathy and certain cancers including breast, colorectal, and cervical (27–29). The role of TGFβ in EMT was first shown 25 years ago (30) and is still widely reported; a keyword search for “TGFβ EMT” returns 19,585 results in PMC (27th Sept 2019). Other stimuli known to induce EMT (Figure 1) include epidermal growth factor (EGF) (31, 32), fibroblast growth factor (FGF) (33), hepatocyte growth factor (HGF) (34, 35), vascular endothelial growth factor (VEGF) (36–38), insulin-like growth factor (IGF) (39, 40), WNT (41), Sonic Hedgehog (SHH) (42, 43), BMPs [BMP-2 (44, 45); BMP-4 (46, 47)], TNF-α (48, 49), and hypoxia (32) with the latter thought to promote EMT via epigenetic regulation of DICER; the enzyme involved in miRNA processing (50).

In recent years, researchers have begun to move away from the idea that only growth factors can stimulate EMT. Novel non-growth factor stimuli, which have been implicated in EMT stimulation, include oxalate and Galectin-8. Oxalate—a routine metabolic by-product—and calcium oxalate—whose deposition (Microcalcification type I) is often seen in benign non-malignant breast tissue (51, 52)—induced EMT both in cultured renal cells and *in vivo* (53, 54). Similarly, oxalate-treated mice presented with highly aggressive undifferentiated mammary tumors and *in vitro* oxalate induced breast epithelial cell proliferation and expression of the tumorigenic gene c-fos (55). Calcium oxalate mediates its effect via activation of p38/MAPK (56), and oxalate-induced EMT could be prevented by activation of nuclear factor erythroid-derived 2-related factor 2 (NRF2) signaling (57). Galectin-8—a widely-expressed glycan binding protein—stimulated partial EMT; tumors arising from Galectin-8 overexpression bore a mesenchymal phenotype whilst still expressing E-cadherin and maintaining cell polarity (58). Mechanistically Galectin-8 activated FAK to transactivate the EGFR and increased expression of matrix-degrading enzymes (uPA, MMP-13) (58). Importantly the authors elude to the potential involvement of other signaling pathways in facilitating Galectin-8 EMT-inducing effects. It is possible that Galectin-8 may interact with calcium signaling to increase FAK—similar to novel pro-EMT TRPV4 signaling (59) (discussed further in the next section)—or it may require vimentin expression in order to increase uPA (60), or perhaps it relies on integrin αVβ3 signaling to activate FAK (61). Loss of the receptor Neogenin 1 (a member of the DCC/Frazzled/UNC-40 family) also induced partial EMT in intestinal epithelial cells, via PI3K and MAPK signaling, and extracellular matrix (ECM)-receptor interactions (62).

Despite the novel EMT stimuli above, there are still unanswered questions. It is possible that there are other seemingly harmless metabolic by-products and binding proteins that stimulate EMT. How oxalate promotes pro-cancerous gene expression, and whether it interacts with HATs or other histone modifying enzymes remain to be investigated. Knowing that these stimuli can induce EMT is just the first step in understanding how non-canonical signals can cause EMT. In addition, deciphering the cellular markers and epigenetic signatures associated with partial EMT is crucial. Despite the similarities in the step wise process (the cadherin switch, for example) and with the endpoint ultimately unchanged, it is conceivable that for example, hypoxia-induced EMT may be different from TGFβ-induced EMT in terms of the underlying epigenetic signature and responsiveness to treatment; Cursons et al. showed that where the primary EMT stimulus was hypoxia, treatment directed cells to acquire a more aggressive mesenchymal phenotype (32). Thus, if we were to develop and evaluate therapeutics that selectively target different EMT stimuli as opposed to common downstream targets, perhaps this could enable us to minimize pathological conditions and/or adverse effects which would translate to prognostic differences in patients. Having said this, pre-clinical research of EMT-specific therapeutics is ongoing and yielding promising data. Regorafenib—a multi-kinase inhibitor pre-approved as a second-line treatment in metastatic CRC—displayed potent anti-EMT effects on invasion and metastasis *in vitro* and *in vivo* by inhibiting TGFβ-induced STAT3 phosphorylation (required for Twist1 and Zeb1 activation) (63).

Antibody-mediated approaches are among the frontrunners in anti-EMT therapy. For example, an anti-MMP9 antibody—which targets an enzyme involved in EMT-mediated ECM remodeling in cancer—increased survival and reduced metastatic burden and the EMT marker vimentin in an *in vivo* model of pancreatic cancer (64). Similarly, in an *in vivo* breast cancer model a monoclonal antibody that blocks active MMP9 inhibited spontaneous and experimentally induced lung metastases (65). Clinical trials using an anti-MMP9 antibody (Adecaliximab) alone or in combination therapies are ongoing. It is hoped that these agents will perform better than previously tested broad-spectrum MMP9 inhibitors, which failed in clinical trials due to dose-limiting side effects (64).

In prostate cancer a monoclonal antibody targeting N-cadherin reduced invasion, metastasis, and proliferation of cancer cells as well as Akt activity (66). However, as N-cadherin is expressed in the heart, peripheral nerves, and liver, adverse off-target effects are a huge concern (66).

It is assumed that upregulation of E-cadherin would be beneficial in cancer, given its downregulation in many cancers. In some instances, this is true; in RCC cell lines small activating RNA stimulated E-cadherin expression and subsequently inhibited cell migration and invasion, and downregulated vital pro-EMT genes such as Zeb1 and vimentin (67). However, E-cadherin was required for invasive ductal carcinoma cell survival and metastasis in multiple *in vivo* models of breast cancer (68), finally explaining why E-cadherin is often observed in patients with breast cancer. These data also highlight the potential pitfalls associated with assumed pro-/anti-EMT markers. In addition, such therapeutics can be limited by poor tissue penetration and short biological half-lives (69), and antibody therapeutics are often very expensive to produce and may not be covered by an individual's health insurance.

Despite copious research papers that detail microRNAs that promote or inhibit cancer development, the translation of these data to tangible microRNA drugs is lacking. While no Phase 3 trials are ongoing in this area, new candidates (like RGLS5579 for glioblastoma) are entering the early phases of clinical trials (70), and targeted inhibition of microRNA-155 and microRNA-21 has successfully resensitized tumors to chemotherapy in lung and breast cancers, respectively (71, 72).

EMT therapeutics that repurpose existing FDA-approved drugs is most exciting, as it significantly reduces the translational time of such therapeutics. A comprehensive bioinformatics analysis of pre-approved drugs has proposed and validated various drug combinations, like IKBK and SRC kinase inhibition together with HDAC inhibition, to hamper EMT (73). Often, scientists researching novel EMT drug candidates focus on preventing an epithelial cells transition to an invasive mesenchymal cell, or inducing MET to revert to a normal epithelial state. However, MET may in fact enhance metastatic outgrowth (74, 75). Recently, Ishay-Ronen and colleagues demonstrated a novel method of preventing cancer progression that exploits the plasticity of cancer cells (76). By transdifferentiating post-EMT mesenchymal cancer cells into functional post-mitotic adipocytes—using a combination of pre-approved drugs (Rosiglitazone and a MEK inhibitor)—primary tumor growth and metastasis was repressed (76). Importantly, epithelial cells were immune to adipocyte transdifferentiation (76), highlighting the specificity of this targeted therapeutic. Other novel EMT therapeutics include *Antrodia salmonea*—a fungus indigenous to Taiwan and known for its anti-cancer properties (77). *In vitro, Antrodia salmonea* significantly reduced invasion and reversed EMT by modulating NFκB and WNT/β-catenin signaling, and *in vivo* treatment with *Antrodia salmonea* reduced breast cancer cell-induced lung metastasis (77).

## In Tune With EMT Intracellular Signaling

**Figure 2:**
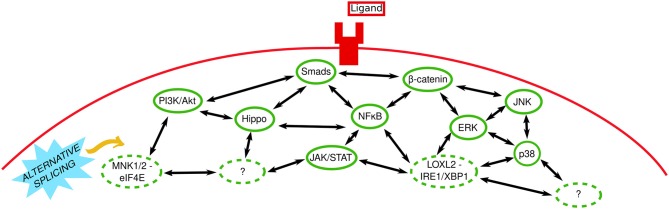
In tune with EMT intracellular signaling. Extracellular stimuli bind to their receptors (shown here as a generic ligand binding a generic transmembrane receptor) and signal via canonical and non-canonical intracellular signaling pathways. Solid and dashed green ovals represent established and novel signaling pathways, respectively. Two-headed black arrows indicate inter-pathway interaction and regulation. Question marks represent unknown pathways involved. The yellow arrow indicates that the role of alternative splicing (sky blue spiked shape) remains to be investigated.

EMT is dependent on the concomitant activity of multiple signaling pathways (Figure 2), which are constantly firing and fluctuating, with turnover time for signaling molecules sometimes infinitesimal [e.g., the half time of HIF-1α clearance is 3–6 min in normoxia (78)]. Positive and negative feedback loops are also incorporated into intracellular signaling pathways, which also rarely work independently and often regulate each other; TGFβ can promote HIF-α expression independent of PI3K by encouraging HIF-1α/HIF-2α translation (79). Downstream TGFβ signaling is the predominant intracellular signaling pathway in EMT. However, as detailed above different stimuli utilize TGFβ signaling and other pathways such as NFκB, YAP/TAZ (Hippo pathway), PI3K/Akt, ERK/JNK, Wnt, Notch, and JAK/STAT3.

While there are copious research papers and reviews detailing the separate roles of these pathways in EMT [see (24, 80–85)], a systems biology approach—or simply data-sharing between groups working on different pathways—would facilitate further elucidation of the intricate nature of signaling pathways and their molecules in EMT. For example, Akt-mediated NFκB activation has been implicated in EMT (86) and our lab performed comprehensive genome-wide analysis of the active open—transcription facilitating—chromatin mark H3K27ac and subsequent gene expression in mammary epithelial cells undergoing EMT (87). We discovered that ERK signaling was essential for epigenetic reprogramming underlying characteristic EMT-related gene expression and phenotypic changes; ERK inhibition prevented the loss and gain of H3K27ac at epithelial and mesenchymal genes (i.e., repression and expression, respectively) (87).

Unraveling inter-pathway regulation in EMT and deciphering what signaling pathways are required for different stages/steps of EMT (e.g., initiation, progression, maintenance, the cadherin switch, loss of cell polarity and other phenotypic alterations) is of vital importance. Recently, the DEPTOR (DEP domain containing mTOR interacting) protein which normally inhibits mTOR signaling, stimulated partial EMT in HCC cells by activating TGFβ-Smad3/4-Snail signaling through mTOR inhibition (88). Similarly, *in vitro* mimicking of ECM organization showed that the way the ECM is arranged could trigger partial or full EMT (89). This process was dependent on YAP activation and subsequent YAP-mediated feedback; Wilms Tumor-1-YAP mediated E-cadherin downregulation, and YAP-TRIO-Merlin-mediated Rho GTPase regulation, which, respectively, loosened cell-to-cell contacts and increased cell migration, compromising epithelial sheet integrity, facilitating EMT, and promoting invasion (89). The location of this phenomenon, i.e., several cells away from the sheet edge, or at the sheet edge itself, determined whether EMT was partial or full, respectively (89). Conversely, single-cell RNA sequencing of untreated epithelial cells vs. those treated with TGFβ showed continuous waves of gene regulation in EMT as opposed to discrete “partial” stages (90). The authors showed that deleting/interrupting key signaling moieties or events can cause cells to build-up at regulatory “checkpoints” that mimic “partial” stages, and enrich a particular pattern of gene expression in said cells which creates the impression of a stable intermediate phenotype (90).

It is also essential that the roles of various signaling pathways—like the aforementioned FAK signaling involved downstream of Galectin-8 (58)—in mediating global transcriptional changes through epigenetic regulation, which are required for EMT, be clarified. That said there are publications that are beginning to decipher these complex interactions. Of note, our laboratory was among the first to report TGFβ and JNK signaling in tandem in EMT; TGFβ signaling via canonical Smads was required for EMT initiation whereas JNK signaling was necessary for EMT to progress to fruition (91). Similarly, an Australian group showed that non-canonical ERK1/2 was needed for initiation but not progression of TGFβ-induced EMT in the rodent eye (92).

The importance of calcium signaling in EMT has gained considerable momentum in recent years, not only because of the discovery of calcium oxalate as a novel EMT stimuli, but because the Ca^2+^-binding protein calreticulin (CRT) was shown to be increased by—and able to regulate—TGFβ-induced EMT *in vitro* (93). Similarly, the TRPV4 ion channel was shown to mediate calcium-dependent downregulation of E-cadherin in breast cancer via activation of Akt signaling (59). Given the fundamental importance of calcium signaling, there are obvious caveats to widespread calcium chelation/calcium channel blockade—similar to widespread DNA methyltransferase (DNMT) inhibition. That said the development of highly specific calcium chelators has the potential to not only reduce EMT but to potentially do so with minimal adverse effects on the patient. The unknown here is whether the beneficial effects on EMT are (a) due to calcium blockade alone or off-target effects on other signaling pathways, and (b) enough to outweigh any pro-EMT effects that may arise from calcium blockade. For example, in addition to reducing E-cadherin TRPV4 activation also augmented FAK phosphorylation (similar to Galectin-8, above), reduced β-catenin and fibronectin-1, and increased expression of the cytoskeletal protein talin-1 (59), an essential protein in maintaining the structural integrity of focal adhesions (94). How TRPV4 induces EMT when it reduces β-catenin and fibronectin, and the key EMT genes TRPV4 regulates to cause EMT without β-catenin and fibronectin, are still elusive.

Non-canonical interactions and lesser-known signaling pathways in EMT are an additional unknown. For example, interactions between TGFβ and the cytoskeleton are emerging (95, 96), with the histone demethylase (HDM) JMJD5 required for cytoskeletal stabilization and inhibition of TGFβ-induced migration in lung cancer cells (95). In terms of lesser-known signaling pathways that are functional in EMT, MNK1/2-eIF4E signaling (97), LOXL2-IRE1/XBP1 signaling (98), and miRNA-20a-FBXL5/BTG3 signaling (99) are examples of seemingly isolated signaling pathways that have recently been implicated in EMT, yet other remote pathways might be involved. Inhibition of MNK1/2-eIF4E signaling using a novel retinamide yielded an impressive trifecta of anti-growth effects, inhibition of EMT, and androgen receptor signaling (97). Overexpressed LOXL2 (lysyl oxidase-like 2) accumulates in the endoplasmic reticulum (ER) where it forces detachment of heat shock protein 5A (HSP5A) from IRE1/XBP1 to activate the IRE1/XBP1 signaling pathway, which confers expression of notable EMT-TFs such as Snail, Zeb2, and Slug in an XBP1-dependent manner (98). However, given the fact that IRE1 inhibition can block XBP1-mediated TF expression (98), more research is needed to decipher this mechanism. FBXL5/BTG3 are direct miRNA-20a targets that are silenced to enable miRNA-20a-mediated EMT and invasion (99), however the precise role of FBXL5 signaling in EMT requires further research; FBXL5 overexpression reduced Snail protein levels (100), but also supported tumorigenesis by negatively regulated PTEN while promoting PI3K/Akt and mTOR phosphorylation and expression (101). The above data on FBXL5 agrees with previous research in our lab, where we discovered that FBXO32—another F-box protein—was pivotal in upstream EMT regulation not only in tumor metastasis, but in neuronal development also (102). FBXO32 directly ubiquitinated CtBP1 causing its stabilization and nuclear retention, which created a suitable microenvironment for EMT progression by facilitating epigenetic remodeling and transcription of CtBP1 target genes (102). In line with these findings, FBXO32 was amplified in metastatic cancers, and its depletion *in vivo* inhibited tumor growth and metastasis (102). These data highlight how important it is for scientists today to challenge pathways that were believed to be futile in EMT as well as those that are routine.

## Factoring in Transcriptional Regulation in EMT

Transcription factors play important roles in cell-fate decisions, and a hierarchy of TF signals may influence whether a cell is epithelial, intermediate, or mesenchymal. Similarly, TFs may operate in a dose-dependent manner, for example, Twist may be required in nanomolar concentrations whereas Snail may be required in micromolar concentrations; indeed, in an EMT time course Twist was transiently detected whereas neither Snail nor Slug were significantly detected (103). Similarly, SNAIL proteins were shown to promote EMT with different potencies in human mammary epithelial cells (104). This is a novel exciting area of research; however, it is beyond the scope of this review. As the intermediaries between cellular signaling and chromatin, these DNA binding proteins guide the epigenetic machinery to their target sites which facilitates chromatin landscape changes at promoter and enhancer elements to drive downstream activation and repression of mesenchymal and epithelial genes, respectively (3, 10). Much is known about the core TFs involved in EMT (Snail, Zeb1/2, and Twist) but a large number of TFs are required for EMT.

**Figure 3:**
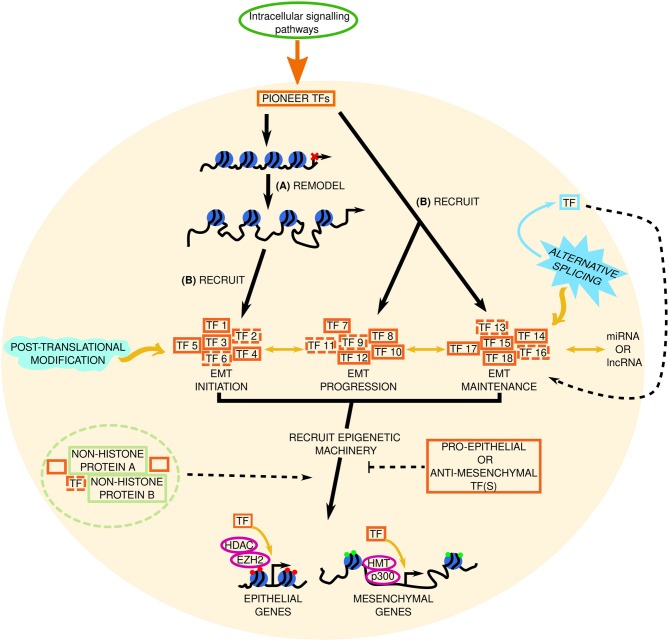
Factoring in transcriptional regulation in EMT. The signal from intracellular signaling pathways is transduced to pioneer transcription factors (TFs) in the nucleus (represented by the beige circle). We hypothesize that in EMT these pioneer TFs: **(A)** remodel the chromatin to increase accessibility at EMT initiating genes, and **(B)** recruit a set of TFs needed for EMT initiation (TF 1–TF 6, dashed lines indicate potentially unknown TFs), and recruit sets of TFs involved in EMT progression (TF 7–TF 12) and maintenance (TF 13–TF 18), respectively, to already accessible chromatin. Through subsequent interactions with epigenetic machinery (such as histone methyltransferases [HMT, e.g., EZH2, enhancer of zeste 2; and enzymes involved in histone acetylation (p300) or deacetylation (histone deacetylase, HDAC)] these TFs repress or promote epithelial and mesenchymal gene expression, respectively, to determine the cell phenotype. Red and green circles at these genes denote repressive or active chromatin marks, respectively. Pro- or anti-EMT TFs may inhibit this. Solid and dashed orange boxes represent established and novel TFs, respectively. Two-headed black arrows indicate inter-pathway interaction and regulation. Yellow arrows and black dashed lines indicate key questions that remain to be investigated. The roles of alternative splicing (sky blue spiked shape) and post translational modifications (mint green wave-like shape) in EMT TF regulation are also elusive. Potential TF-non-histone protein complexes are found in the light green dashed circle. lncRNA, long non-coding RNA; miRNA, micro RNA.

A vast amount of EMT research has focused on TF involvement in the latter stages of EMT, but until recently, little was known about the transcriptional networks that trigger EMT. Newly published data shows that EMT involves a temporal hierarchy of collaborative transcriptional networks (105). This predicted network operates between TFs and between TFs and microRNAs and is composed of 46 (co)transcription factors and 13 miRNAs that were critically required for EMT in NMuMG cells (105). We are also beginning to decipher the requirement of different TFs for EMT initiation, maintenance, and progression (Figure 3). For example, the TF NRF2 delays the transition of a cell to a full mesenchymal phenotype; it maintains the hybrid epithelial/mesenchymal (partial/intermediate EMT) state (106). Similarly, research from our lab has shown that JNK-induced TFs and subsequent signaling are not required for EMT initiation, but was essential for progression of phenotypic hallmarks of EMT (91). Our lab has identified novel JNK-induced TFs that are required for EMT, are highly expressed in invasive cancer cells, and induced during neuronal development (91). These data are pivotal in the fight against EMT because the authors do not examine these novel TFs individually in EMT; they also define their role(s) in EMT that occurs in neuronal development.

An unknown in this area, is the existence of TFs that do more than just control gene expression—perhaps TFs can perform secondary functions, such as chromatin remodeling (Figure 3), or mediate epigenetic regulation in EMT. In terms of TFs that exhibit auxiliary properties, our lab identified NeuroD1 as a pioneer factor; it recognizes and binds to its target sites and drives transcription of neuronal/EMT-related genes (such as Hes6 and Dll3, Snai2 and Twist1, respectively) regardless of the chromatin state (open or closed) or cell type (e.g., ESCs and differentiated fibroblasts) (107, 108). NeuroD1 is not alone in its pioneer TF abilities; FOXA TFs (FOXA1, FOXA2, FOXA3) (109, 110) and GATA3 (109) are also pioneer factors that determine cell fate. Heat shock factor 1 (HSF1) which induces HSPs is another pioneer factor with a secondary function in recruiting DNMT3A to suppress miRNA-137 and promote carcinogenesis (111), and perhaps HSF1 is also involved in HSP5A-mediated regulation of LOXL2-IRE1/XBP1 signaling. Despite these advances, the specific involvement of pioneer TFs in EMT in cancer remains to be seen.

TF that mediate epigenetic alterations are coming to the forefront of the field; Zeb2 employs DNA methylation to repress RAB25—a small Rab GTPase with a potential role in epithelial polarity—and E-cadherin, and increased SIRT-1-mediated H3K9 deacetylation at both promoters to maintain this suppression (112). FOXD3 both permits and prevents H3K27ac of regulatory elements and acts as a pioneer factor during neural crest (NC) development to prime NC factors (e.g., *snai1b, twist1b*), acts upstream of pro-EMT factors, and during NC EMT FOXD3 mediates a cadherin switch (decreased N-cadherin and increased cadherin 7) to modulate cell adhesion (113, 114). BPTF (bromodomain PHD finger TF), a TF which is also the largest subunit of the NURF chromatin remodeling complex [a member of the ISWI remodeling family (11, 115)], was increased in HCC where it inferred poor survival and correlated with high vimentin and low E-cadherin expression (116). These data show us that TFs are more than a “one trick pony”—they can influence the epigenetic signature of a cell. It was accepted that all relevant EMT-TFs had been identified but given the discovery of novel EMT-TFs in recent years and the fact that we still can't resolve EMT, perhaps there are unknown EMT-TFs (capable of altering a cells epigenetic state) and unknown ENT-TF targets that are yet to be discovered. Sox4 was known to play a role in EMT (117), but it was not until the following year that its direct transcriptional targets—like EZH2, fibronectin, N-cadherin—and role as a master regulator of EMT were elucidated (118). EZH2 in turn suppresses the tumor suppressor hepatocyte nuclear factor 1β (HNF1B) which normally represses Slug (119). Similarly, Sox8-mediated chemoresistance and stemness in tongue squamous cell carcinoma was only recently shown to involve Frizzled-7/Wnt/β-catenin signaling (120).

Perhaps alternative splice isoforms of existing TFs are yet to be discovered. For example, splice variant 1 (SV1) is a truncated isoform of KLF6 that has no zinc finger domains, yet it promotes tumor migration, and is thought to stimulate EMT in HCC (121). Hepatocyte nuclear factor 4α (HNF4α) has alternative isoforms that both stimulate (HNF4α7, 8, 9) and repress (HNF4α1, 2, 3) cancer development (121–123). We also cannot rule out the possibility of alternative TF splice isoforms in epithelial and mesenchymal cells. Their existence and how they influence cell signaling in EMT remain elusive. Indeed, extracellular ligands, DNA/histone modifying enzymes, and chromatin remodeling proteins may also be subjected to alternative splicing in EMT. Non-TF proteins could also be subjected to alternative splicing, to enable pro-EMT TFs. Recently, exon skipping in FLNB (an actin-binding protein) was shown to induce EMT by releasing the pro-EMT TF FOXC1 (124). This alternatively spliced isoform of FLNB also correlated with EMT gene signatures in basal breast cancer samples (124).

In addition, there may be TFs that we believe are fully elucidated, and so are not examined in the context of EMT. For example, GLI TFs are the primary mediators of developmental Hedgehog-GLI signaling pathway, which is mostly inactive in adults (except for in stem cells) (125), but GLI inhibition was found to block EMT in pancreatic cancer stem cells through reversion of the typical EMT cadherin switch and blockade of EMT-TFs (126). Together these data make the HH-GLI pathway an attractive—previously unthought-of—target in EMT therapy and add to the rationale for examining TFs in other developmental pathways, but it has taken over a decade for such pathways to become a focus of therapeutics—which is perplexing given that TFs necessary for development are often implicated in EMT.

We also cannot rule out the possibility of TFs forming fixed complexes with epigenetic proteins or non-histone proteins in EMT. For example, the non-histone chromatin protein HMGA1 forms a complex with the TF FOXM1 which stabilizes FOXM1 in the nucleus and increases expression of shared target genes like VEGFA to promote angiogenesis, inferring a negative prognosis (127). This is not the only HMG protein implicated in EMT; the pro-EMT role of HMBG-1 is discussed below. Perhaps TFs are epigenetically altered in EMT to silence or amplify their effect. Take MYC-GATA3/ESR1 TFs for instance; MYC overexpression increased MYC enrichment and reduced active histone marks (H3K27ac, H3K4me1/3) at regulatory elements for GATA3 and ESR1, resulting in cellular dedifferentiation i.e., a stem cell-like state (128). These data were validated in breast cancer patients where there was an inverse relationship between MYC overexpression and GATA3/ESR1 gene levels (128). It is plausible that there are other TFs that interact like this in EMT.

## Epigenetic Mechanisms Involved in EMT—Room to Explore

Histones are the primary component of chromatin. Positioning DNA around histone octamers—nucleosomes—to form chromatin is essential to maintain the integrity of the genome; chromatin prevents DNA strand entanglement, but also dynamically regulates DNA replication and gene expression (129, 130). Extracellular stimuli, intracellular pathways, and transcription factors are subject to variation between patients and between cancers, and chromosomal instability promotes cancer (131), but the DNA is the same in all cells of the organism (132) and mechanisms and regulators of chromatin condensation are well-known (133). This makes chromatin the ultimate platform for action. Epigenetic—“above genetic”—modifications alter the genetic read-out of a cell, and include DNA methylation, histone modifications, and chromatin higher order structures (such as looping), which in collaboration with chromatin remodelers determine chromatin accessibility.

Establishing the epigenetic state involves epigenetic readers, writers, and erasers which recognise, create, or remove epigenetic modifications respectively (134). A patient's epigenetic state is therefore both dynamic and stable, and is involved not only in disease pathogenesis but in their response to treatment and their rates of disease-free survival and recurrence (135–138). Therefore, epigenetic regulators are prime therapeutic targets, and understanding epigenetic mechanisms of regulation would pave the way for novel therapeutics and personalized cancer medicine. We know that EMT-TFs guide the epigenetic machinery to target promoters/enhancers, but we do not know the mechanism behind this; i.e., how cells decide which loci are targeted during EMT initiation, progression, and maintenance.

Single-cell analysis has shown that during development progenitor cells display epigenetic heterogeneity (139). Specifically, the pre-EMT and delaminating crest (undergoing EMT) generate migrating progenitor cells whose heterogeneity is associated with transcriptional properties of early genes from competing downstream cell fates, which are activated when NC cells undergo EMT (139). The authors also note that mesenchymal potential may be established as early as delamination; lone overexpression of *Twist1* was able to drive trunk NC toward a mesenchymal fate rather than the traditional neuronal fate (139). These data provide us with a better understanding of developmental NC EMT which in turn aids our understanding of NC-derived cancers such as melanoma and glioma, and perhaps such heterogeneity is present in other cancers (139, 140).

**Figure 4:**
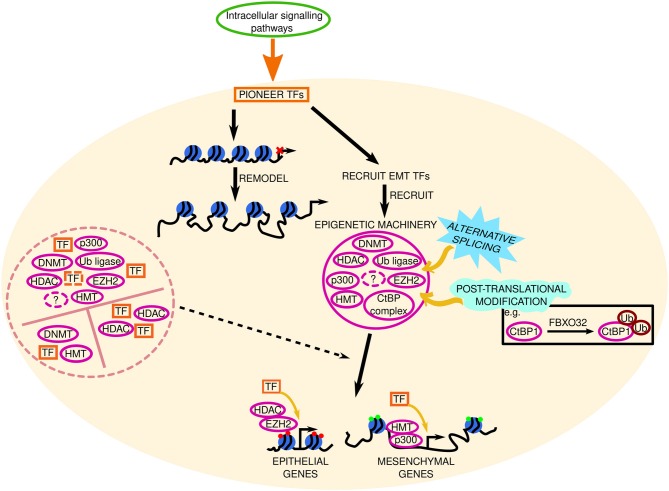
Epigenetic mechanisms involved in EMT. The signal from intracellular signaling pathways is transduced to pioneer transcription factors (TFs) in the nucleus (represented by the beige circle). These pioneer TFs remodel the chromatin and recruit EMT TFs to accessible chromatin, which subsequently recruit and interact with epigenetic machinery (solid pink circle), such as DNA methyltransferases (DNMT), histone methyltransferases (HMT, e.g., EZH2, enhancer of zeste 2; ubiquitinating enzymes, Ub ligase; CtBP, C-terminal binding protein; complex, and enzymes involved in histone acetylation (p300) or deacetylation (histone deacetylase, HDAC) to target loci. Together, TFs and epigenetic machinery repress or promote epithelial and mesenchymal gene expression, respectively, to determine the cell phenotype. Red and green circles at these genes denote repressive or active chromatin marks, respectively. Solid and dashed pink circles represent established and novel epigenetic machinery, respectively. Black dashed lines indicate key questions that remain to be investigated. The roles of alternative splicing (sky blue spiked shape) and post translational modifications (mint green wave-like shape) in EMT epigenetic machinery are also elusive. FBXO32 is an ubiquitin ligase. Question marks represent unknown epigenetic machinery. Potential TF-epigenetic machinery complexes are contained within the pink dashed circle.

Epigenetic regulation of EMT (Figure 4) has mainly focused on histone methylation/acetylation, with additional studies detailing the roles of other histone modifications such as phosphorylation (141–143), ubiquitination (144–146), citrullination (85, 147–149), SUMOylation (150–152), and biotinylation (153) in EMT. Histone modifying enzymes are dynamic and diverse. Established enzymes [reviewed in (3, 10)] are involved in DNA methylation (DNMTs), histone (de)methylation (HMTs, HDMs), histone (de)acetylation (HATs, HDACs), and ubiquitination (E3 ubiquitin ligases). Despite the wealth of knowledge regarding these enzymes, we have much to learn. For example, class I HDAC enzymes generally promote tumorigenesis, and class IIA may promote or impede cancer development; however it was only recently discovered that HDAC5 induced anti-proliferative or pro-EMT effects depending on the cell line in which it was overexpressed (154).

Unsurprisingly a number of these enzymes contain methyl-lysine binding motifs, which curiously differ between enzymes; DNMT3A contains a PWWP domain to read H3K36me3 (155), ankyrin repeats in the histone methyltransferase (HMT) G9a enable H3K9me1/2 binding (156), and a double Tudor domain exists in the HDM JMJD2A for H3K4me3 and H4K20me3 binding (157). Even though these binding motifs are crucial for correct chromatin recognition—needed for enzymatic effects on chromatin methylation state—the importance of these different domains in EMT remains unknown; we do not know why there are three distinct methyl-lysine binding motifs among histone modifying enzymes, or whether they play a causal role in EMT development or not.

PWWP domains are distinct from the catalytic domain in DNMT3A (155). Missense mutations in the PWWP domain (W330R) abolish H3K36me2/3 binding and cause DNA hypermethylation at Polycomb-regulated regions in patients and *in vivo* (158). This is associated with reduced H3K27me3 and H3K4me3 bivalent marks, and modified chromatin accessibility at key developmental regulators (e.g., SOX and HOX family members, FOXA1, NEUROG1/2) (158). The implications for such mutations in EMT are still unknown. Perhaps mutations in the PWWP domain of DNMT3A confer increased methylase activity and allow the mutant to methylate polycomb regions, thus preventing PRC2 binding and EZH2-mediated H3K27me3 at epithelial genes as suggested by Heyn et al. (158). Alternatively, maybe methylase or other DNA recognition/binding motif activity is decreased upon PWWP mutation, allowing mesenchymal gene expression. The PWWP domain might even interact with non-histone proteins in EMT; however whether this is strengthened or weakened in mutant DNMT3 remains to be investigated. Whether mutant DNMT3A has enhanced or diminished recruitment by the aforementioned pioneer factor HSF1 also remains to be seen. Studies that examine human cancers for the presence of these and other PWWP mutations would be worthwhile, as the PWWP binding motif is highly conserved in human hepatoma-derived growth factor (HDGF) which is overexpressed in a number of human cancers and is involved in PI3K signaling (159). We cannot rule out a causative—or at the very least a contributing—role for PWWP domain mutations in EMT development in cancer, thus deciphering the unknowns that still surround the PWWP domain is of great importance.

The methyl-lysine binding motif in the HMT G9a contains ankyrin repeats; the rationale behind ankyrin repeats over a PWWP domain is unknown. Perhaps each motif bestows different effects on epigenetic regulation of EMT. In EMT G9a interacts with Snail which recruits it to the CDH1 promoter (160). Enhanced in hypoxia, G9a activity is beneficial to cancer cell survival and G9a can methylate non-histone proteins (161), like the ATPases Pontin and Reptin (162). The functional importance of the ankyrin repeats was only recently discovered. Levels of H3K9 mono/di/tri-methylation were analogous in ESCs expressing either wild-type G9a, G9a bearing a mutation in ankyrin repeats, or G9a lacking ankyrin repeats (163). However, the HMT activity of G9a/GLP is dependent on the ankyrin repeats (164). These data illustrate that these enzymes are more than just their catalytic activity. In terms of G9a-Snail interactions in EMT, the role of the ankyrin repeats in facilitating non-histone protein (e.g., E-cadherin) methylation required elucidation. It is plausible that there are G9a variants—like the variant that skips exon 10 (165)—that are alternatively spliced in exons pertaining to the ankyrin repeats, which may be present and causal in EMT. In line with this, a novel splice isoform of the arginine methyltransferase PRMT1 which lacks exons 8/9 (that encode for the dimerization arm, essential for enzymatic activity) was increased in cancer cell lines and induced by Snail (166). Similarly, PRMT9 elicited pro-invasive and metastatic effects via Snail and the PI3K/Akt pathway, which was validated in clinical HCC samples (167). Given the high stability of histone modifications in bodily fluids (168), methylated arginine residues could be developed as a novel prognostic biomarkers/therapeutic targets.

The role of Tudor domains (found in the HDM JMJD2A) in epigenetic regulation of EMT is yet another unknown. In addition to recognizing and binding H3K4me3 and H4K20me3, the two tandem Tudor domains in JMJD2A bind H3K9me3 and H4K20me2 which confer roles for JMJD2A in initiating and preventing transcription (169). As there are two Tudor domains, these conflicting functions might be localized to a specific domain, or the two Tudor domains might have opposing effects on EMT. Further work is required to determine whether JMJD2A has a pro- or anti-EMT effect given its ability to silence *and* active genes. Given the aforementioned involvement of fellow Jumonji family member JMJD5 in inhibition of TGFβ-induced migration in lung cancer cells (95), it is plausible that JMJD2A has a similar anti-EMT effect, however this claim warrants further research. In addition, to stabilize chromatin binding and mediate its epigenetic effects—and oncogenic effects—JMJD2A must be SUMOylated at K471 (170). We know that lysine residues can accept different epigenetic modifications, but there is no hard and fast rule that the enzymes that perform these histone/DNA modifications are exempt from modification themselves; For example, phosphorylation of G9a on Ser569 is crucial for its recruitment to damaged DNA (171).

## Remodeling Our Understanding of Chromatin Machinery in EMT

Chromatin remodeling can involve nucleosome repositioning, swapping, displacement or even translocation to a fragment on a neighboring strand of DNA, as well as histone replacement (11, 172). In addition to roles in nucleosome positioning and DNA dependent biological processes such as repair and replication (173), remodelers are crucial determinants of chromatin accessibility and subsequent gene expression (11). Four major chromatin remodeling families are characterized; SWI/SNF, ISWI, CHD, and INO80 (11). Genes encoding components of SWI/SNF (e.g., BRG1) are among the most common targets of mutation in cancer; ~20% of human tumors were shown to contain mutations in at least one member of this complex (11). Despite this alarming statistic, mechanisms of epigenetic regulation by chromatin remodeling proteins and histone chaperones—in particular, how such regulation may be detrimental to our health, as is the case in EMT-related tumorigenesis—are largely overlooked.

**Figure 5:**
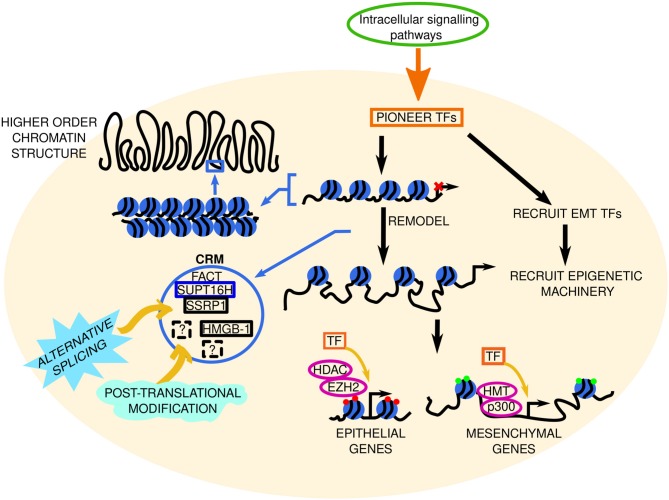
Chromatin remodeling machinery in EMT. The signal from intracellular signaling pathways is transduced to pioneer transcription factors (TFs) in the nucleus (represented by the beige circle). These pioneer TFs remodel the chromatin and recruit EMT TFs to accessible chromatin, which subsequently recruit and interact with epigenetic machinery such as DNA methyltransferases, DNMT; histone methyltransferases, HMT; e.g., EZH2, enhancer of zeste 2; ubiquitinating enzymes, Ub ligase; CtBP, C-terminal binding protein; complex, and enzymes involved in histone acetylation (p300) or deacetylation (histone deacetylase, HDAC). Together, TFs and epigenetic machinery repress or promote epithelial and mesenchymal gene expression, respectively, to determine the cell phenotype. Red and green circles at these genes denote repressive or active chromatin marks, respectively. The function of higher order chromatin structures in EMT phenotype determination remains elusive. Chromatin remodeling machinery (CRM) within the blue circle control chromatin accessibility and gene expression. Solid and dashed black boxes represent established and novel CRM, respectively. FACT, facilitates chromatin transcription; composed of SSRP1, structure specific recognition protein 1; and SUPT16H (SPT16 homolog, facilitates chromatin remodeling subunit); HMGB-1, high mobility group box 1. Question marks represent unknown CRM. The solid blue box around SUPT16H signifies that this CRM has PH domains, whose role in EMT is unknown. The roles of alternative splicing (sky blue spiked shape) and post translational modifications (mint green wave-like shape) in EMT CRM are also elusive.

Known chromatin remodelers with functional roles in EMT (Figure 5) have been reviewed see (174) and include the NuRD subunits MTA1 and MTA3 within the CHD family, the BPTF subunit of the ISWI family member NURF (116), and the BAF250/ARID1, BRG1, and hBRM subunits of the mammalian SWI/SNF complex (174, 175). Interestingly, the combined loss of BAF250A/ARID1A and gain of expressed mutated PI3K subunit PIK3CA^H1047R^ results in partial EMT in the endometrial epithelium (176). Resistance to ER antagonists in breast cancer was recently attributed to loss of ARID1A, which reduced HDAC1 activity and increased H4K acetylation—sensitizing the cancer cells to BRD4 inhibition (177). Nucleophosmin 1 (NPM1) is another well-studied histone chaperone [reviewed by (178)] with a role in EMT/invasion (179–181).

In terms of nucleosome structure, the FACT (FAcilitates Chromatin Transcription) histone chaperone complex is a heterodimer consisting of SSRP1 and SUPT16H that has established roles not only in nucleosome destabilization and formation, but in the replacement of histones with histone variants, and FACT also prevents unwanted histone movement during transcription (182). FACT subunits were shown to be overexpressed in breast cancer and pharmacological entrapment of FACT within chromatin—which reduced FACT activity—had positive (dose-dependent) effects on murine survival, prevented tumor onset and delayed progression *in vivo* (183). Given these data, our understanding of the EMT-promoting effects of the chromatin remodeling machinery is lacking. In human intestinal cell lines the FACT subunit SUPT16H positively correlated with epithelial markers such as E-cadherin and showed an inverse relationship with the expression of classical mesenchymal markers like Zeb1 (3). Crystallographic examination of the FACT subunit Spt16 [isolated from yeast, the middle domain (-M) in particular] showed that a double pleckstrin homology (PH) domain exists in Spt16-M (184). Although analogous studies with SUPT16H are required, these data suggest that like its homolog, SUPT16H may contain PH domain(s) to bind to proteins other than histones or to facilitate intracellular signaling. Elucidating the role of PH domains in chromatin remodeling proteins could uncover previously unknown functions of histone chaperone proteins in EMT—their role in regulating chromatin accessibility may be just one avenue through which they promote EMT. Spt16-M could bind to H3-H4 (184), and therefore may influence the methylation/acetylation state of key lysine residues on H3-H4, possibly through interacts with DNMT3A, G9a, JMJD2A, or other epigenetic modifying enzymes, which may increase chromatin accessibility at epithelial gene promoters/regulatory elements and vice versa for mesenchymal genes. The second FACT subunit SSRP1-M was initially thought to lack histone binding (185), however it was later shown to bind H3-H4 as well as H2A-H2B (186). *In vivo* the SSRP1 subunit enhanced xenograft tumor growth/proliferation and SSRP1 overexpression *in vitro* promoted EMT (187). Mechanistically, miRNA-28-5p was implicated in upstream negative regulation of SSRP1 (187), however given the discovery of PH domains within Spt16 and SUPT16H-H3/H4 binding, it is reasonable to suggest that SSRP1 promotes EMT via epigenetic regulation of key genes, albeit further research is required.

The histone chaperone SUPT6H was found to be indispensable in estrogen-dependent breast cancer cells in terms of maintaining chromatin structure, facilitating estrogen-mediated transcription, and suppressing H3K27me3 on lineage-specific genes (188). However, *in vitro* murine Spt6 was shown to offset H3K27me3 by facilitating the H3K27 demethylase KDM6A (a.k.a. UTX, another Jumonji family member) (189). Consequently, Spt6 promoted differentiation and muscle gene expression in skeletal muscle cells (189). UTX was later shown to epigenetically repress the EMT-TFs Snail and Zeb1/2—independent of H3K27 demethylation—via decreased H3K4me2 and H3 acetylation at their promoters (190), with these anti-EMT effects in agreement with previously described data on JMJD5. In contrast, KDM5B overexpression increased cell proliferation, positively correlated with EMT markers, and promoted aggressive tumors in lung cancer (191). These data highlight just one facet of the intricate relationship between chromatin remodeling machinery and epigenetic regulation and illustrate the need for further research into how chromatin remodeling proteins may contribute to EMT.

HMGB-1 (high-mobility group box 1); a well-studied chromatin-binding nuclear protein that acts as a chromosome guardian/DNA chaperone and has immune/inflammatory functions [all of which have been extensively reviewed by (192)], was shown to promote EMT by upregulating MMPs (-1/-3/-10) via RAGE/NFκB pathway (193), and activating the TLR4/p38/NRF2 pathway to facilitate HMGB-1-mediated downstream EMT signaling (194). However, the epigenetics regarding HMGB-1 are unknown. Perhaps HMGB-1 itself epigenetically regulated, given the fact that its promoter coincides with a CpG island (195). Maybe HMGB-1 influences the epigenetic regulation of EMT target genes (epithelial or mesenchymal), or its DNA chaperone activity could promote/prohibit potential epigenetic regulation of target genes by competing with other chromatin proteins for the same chromosomal DNA sites as suggested by Spada et al. (196). Its EMT-promoting effects are somewhat localized to targeted interaction with its 3′ untranslated region (197, 198)—whether the crucial roles of 3′ UTRs in translation efficiency, mRNA stability and subcellular localization (199) play a role in HMGB-1-induced EMT remains to be studied. Given its ubiquitous expression it is possible that HMGB-1 is merely a ticking time bomb which can silently instigate EMT via a multitude of signaling pathways under the guise of other stimuli; HMGB-1 can signal via numerous receptors including but not limited to TLRs, RAGE, and integrins (192), so perhaps the EMT-inducing effects of HMGB-1 can be mirrored by other stimuli that activate these receptors and/or these downstream signaling cascades.

Chromatin Assembly Factor 1 (CAF1) consists of three proteins—p150, p60, and p48—and is necessary for preserving chromatin structure in our cells (200, 201). A novel role for CAF1 in EMT was recently uncovered, where CAF1 was implicated in cellular invasion and motility, and siRNA-mediated CAF1 depletion (specifically the p150 subunit) promoted the development of a Slug and/or claudin-guided EMT-like phenotype (202). Given its primary role as a histone chaperone, CAF1 may facilitate EMT via alterations in chromatin remodeling at key EMT genes, i.e., its depletion may enhance accessibility at mesenchymal genes. While these data implicate CAF1 *depletion* in cancer development, in a protein complex each subunit needs to be studied separately first and then as a whole. The p150 subunit was highly expressed in HCC tissue and epithelial ovarian cancer where it was associated a poor prognosis (203, 204). Similar data were obtained regarding the p60 subunit in prostate and lung cancers (205, 206), however virtually no information is available on p48, as it has not yet been identified in humans. As informative as these data may be, they do not address the gaping hole of how and why these CAF1 subunits are involved. Surely, there are epigenetic mechanisms involved, but as of right now these are unknown.

## Conclusion

**Figure 6:**
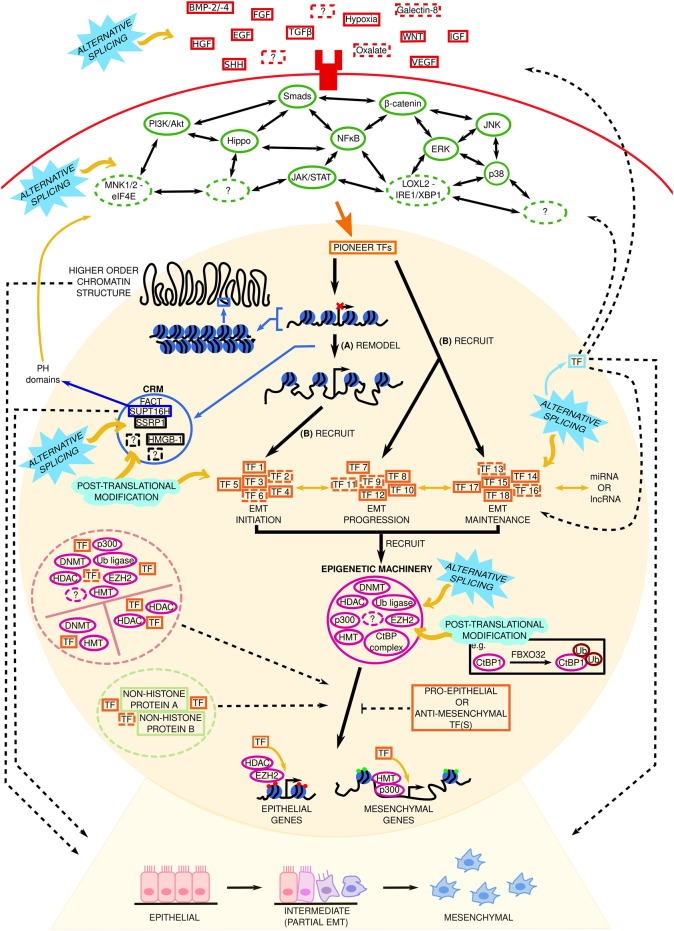
Complexity surrounding the gene regulatory network in Epithelial Mesenchymal Transition (EMT). Established (solid red boxes) and novel (dashed red boxes) extracellular stimuli bind to their particular receptors to initiate EMT. A generic transmembrane receptor is shown here for illustrative purposes. Extracellular stimuli subsequently signal via canonical and non-canonical intracellular signaling pathways. Solid and dashed green ovals represent established and novel signaling pathways, respectively. Two-headed black arrows indicate inter-pathway interaction and regulation. The signal from these signaling pathways is transduced to pioneer transcription factors (TFs) in the nucleus (represented by the beige circle). We hypothesize that in EMT these pioneer TFs: **(A)** remodel the chromatin to increase accessibility at EMT initiating genes, and **(B)** recruit a set of TFs needed for EMT initiation (TF 1–TF 6, dashed lines indicate potentially unknown TFs), and recruit sets of TFs involved in EMT progression (TF 7–TF 12) and maintenance (TF 13–TF 18), respectively, to already accessible chromatin. Solid and dashed orange boxes represent established and novel TFs, respectively. Through subsequent interactions with epigenetic machinery such as histone methyltransferases, HMT; e.g., EZH2, enhancer of zeste 2; and enzymes involved in histone acetylation (p300) or deacetylation (histone deacetylase, HDAC) at target loci, these TFs repress or promote epithelial and mesenchymal gene expression, respectively, to determine the cell phenotype (epithelial, intermediate, or mesenchymal). Red and green circles at these genes denote repressive or active chromatin marks, respectively. Pro- or anti-EMT TFs may inhibit this. Potential TF-non-histone protein complexes are found in the light green dashed circle. lncRNA, long non-coding RNA; miRNA, micro RNA. Solid and dashed pink circles represent established and novel epigenetic machinery, respectively. Black dashed lines indicate key questions that remain to be investigated. FBXO32 is a ubiquitin ligase. Potential TF-epigenetic machinery complexes are contained within the pink dashed circle. The function of higher order chromatin structures in EMT phenotype determination remains elusive. Chromatin remodeling machinery (CRM) within the blue circle control chromatin accessibility and gene expression. Solid and dashed black boxes represent established and novel CRM, respectively. FACT, facilitates chromatin transcription, composed of SSRP1 (structure specific recognition protein 1) and SUPT16H (SPT16 homolog, facilitates chromatin remodeling subunit); HMGB-1, high mobility group box 1. The solid blue box around SUPT16H signifies that this CRM has PH domains, whose role in EMT is unknown. The roles of alternative splicing (sky blue spiked shape) and post translational modifications (mint green wave-like shape) at the indicated steps/components remains to be investigated Question marks represent unknowns at each stage of EMT regulation. Yellow arrows and black dashed lines indicate key questions that remain to be investigated (see main text for more information).

EMT is a dynamic multifaceted process occurring during development, tissue/organ repair, and disease, involving a vast network of signaling molecules working in unison and/or against one another (Figure 6). Just because there are different sub-types of EMT, does not necessarily mean that the stimuli, intracellular signaling pathways, TFs, epigenetic mechanisms, and chromatin remodeling machinery underlying developmental EMT, wound healing/fibrotic EMT, and cancer-related EMT are dissimilar. There must be some common ground between the sub-types and between partial and full EMT. Moreover, the partial/intermediate phenotype is of particular interest given recent single-cell sequencing data depicting continuous waves of gene regulation in EMT as opposed to discrete “partial” stages.

Novel stimuli and intracellular pathways, undiscovered TFs and DNA/histone modifying enzymes, and the role of chromatin remodelers in EMT are huge unknowns in EMT. Auxiliary TF functions and the role of different sets of TFs involved in EMT (initiation versus progression etc.) warrant further investigation, and inter- and intra-tumor epigenetic heterogeneity and the impact of a cancer patient's epigenetic state are other compelling areas of research. To date, the majority of EMT research has focused on individual sub-types; data examining them as one process that occurs at three distinct “time points” is lacking. The challenge for future research therefore lies in (a) examining EMT concomitantly in disease and non-disease states, (b) looking at the interplay between canonical and non-canonical stimuli, TFs, and pathways involved in EMT, and (c) elucidating the roles the chromatin remodeling machinery and alternative splicing in phenotype determination during EMT, all with the aim of unraveling the complexity of the gene regulatory network underlying EMT. The data highlighted in these sections illustrate that researchers have begun to challenge these unknowns, but more work is needed to expand our knowledge and ultimately advance our ability to cure cancer.

## Author Contributions

DL and VT worked on the conceptual framework and generated the final version for submission.

## Conflict of Interest

The authors declare that the research was conducted in the absence of any commercial or financial relationships that could be construed as a potential conflict of interest.

## Abbreviations

AKT: Protein kinase B
ARID1: AT-rich interaction domain 1
BMP: bone morphogenetic protein
BPTF: bromodomain PHD finger TF
BRD4: bromodomain containing 4
BRG1, Brahma-related gene 1, a.k.a. SMARCA4, SWI/SNF related, matrix associated, actin dependent regulator of chromatin, subfamily a: member
BTG3: BTG anti-proliferation factor 3
CAF1: chromatin assembly factor 1
CHD(1): chromodomain helicase DNA-binding (protein 1)
CRT: calreticulin
DCC: deleted in colorectal cancer
a netrin receptor
DEPTOR: DEP domain containing mTOR interacting protein
DNA: deoxyribonucleic acid
DNMT: DNA methyltransferase
ECM: extracellular matrix
EGF: epidermal growth factor
EMT: epithelial to mesenchymal transition
EGFR: epidermal growth factor receptor
EIF4E: eukaryotic initiation factor 4E
ER: endoplasmic reticulum
ERK: extracellular signal-regulated kinase
ESR1: estrogen receptor alpha
EZH2, enhancer of zeste 2: PRC2 subunit
FACT, facilitates chromatin transcriptionFAK: focal adhesion kinase
FBXL5: F-box and leucine rich repeat protein 5
FGF: fibroblast growth factor
FLNB: filamin B
FOX, Forkhead box (A1, C1, D3: M1 denote individual proteins)
GATA3/4, GATA binding protein (3: 4 denote different proteins)
GLI: glioma-associated oncogene
HAT(s): histone acetyltransferase(s)
hBRM, human paralog of *Drosophila* Brahma, a.k.a. SMARCA2 SWI/SNF related, matrix associated, actin dependent regulator of chromatin, subfamily a: member 2
HCC: hepatocellular carcinoma
HDAC(s): histone deacetylase(s)
HDGF: hepatoma-derived growth factor
HDM(s): histone demethylase(s)
HGF: hepatocyte growth factor
HH: hedgehog
HIF-1α: hypoxia inducible factor 1α
HIF-2α: hypoxia inducible factor 2α
HOX: homeobox
HMGA1: High Mobility Group AT-Hook 1
HMGB-1: high mobility group box 1
HMT(s): histone methyltransferase(s)
HNF4α: hepatocyte nuclear factor 4α
HSF1: heat shock factor 1
HSP5A: heat shock protein 5A
IGF: insulin-like growth factor
IRE1: Inositol-requiring enzyme 1
INO80: INO80 complex ATPase subunit
ISWI: Imitation SWItch
JAK: Janus kinase
JMJD2A, Jumonji-C (JmjC) domain-containing protein 2A: a.k.a. KMD4A
JMJD5, Jumonji-C (JmjC) domain-containing protein 5: a.k.a. KDM8
JNK: c-Jun N-terminal kinase
KDM4A: lysine demethylase 4Aα
KDM6A, lysine demethylase 6A: a.k.a. UTX histone demethylase
KDM8: lysine demethylase 8
KLF6: Krueppel-like factor 6
lncRNA: long non-coding RNA
LOXL2: lysyl oxidase-like 2
MET: mesenchymal-epithelial transition
miRNA(s): micro RNA(s)
MNK1/2: mitogen-activated protein kinase-interacting kinases 1 and 2
MTOR: mechanistic/mammalian target of rapamycin
NC: neural crest
NEUROD1: neuronal differentiation 1
NEUROG1: neurogenin 1
NEUROG 2: neurogenin 2
NFκB: nuclear factor kappa-light-chain-enhancer of activated B cells
NPM1: nucleophosmin 1
NRF2: nuclear factor erythroid-derived 2-related factor 2
NuRD: nucleosome remodeling and deacetylase
NURF: nucleosome remodeling factor
MYOSLID, myocardin-induced smooth muscle lncRNA: inducer of differentiation
MAPK: mitogen activated protein kinase
MMP: matrix metalloproteinase
MRNA: messenger ribonucleic acid
MTA1: metastasis-associated antigen 1
MTA3: metastasis-associated antigen 3
PAF: PCNA clamp associated factor
PCG: Polycomb group
PH: pleckstrin homology
PI3K: phosphoinositide 3-kinase
PIK3CA, phosphatidylinositol-4: 5-bisphosphate 3-kinase catalytic subunit alpha
PTEN: phosphatase and tensin homolog
PRC2: polycomb repressive complex 2
RAGE, a.k.a. AGER: advanced glycosylation end-product specific receptor
RNA: ribonucleic acid
SHH: sonic hedgehog
SIRT-1: Sirtuin 1
SOX4: SRY-box transcription factor 4
SSRP1: structure specific recognition protein 1
STAT3: signal transducer and activator of transcription 3
SUPT6H, SPT6 homolog: histone chaperone and transcription elongation factor
SUPT16H, SPT16 homolog: facilitates chromatin remodeling subunit
SWI/SNF: SWItch/Sucrose Non-Fermentable
TAZ: transcriptional coactivator with PDZ binding motif
TF(S): transcription factor(s)
TGFβ: transforming growth factor beta
TLR4: Toll-like receptor 4
TRIO: Rho guanine nucleotide exchange factor
TRPV4: transient receptor potential vanilloid 4
UNC-40, un-coordinated 40: a netrin receptor. *C. elegans* homolog of DCC
UPA: urokinase-type plasminogen activator
UTRs: untranslated regions
VEGF(A): vascular endothelial growth factor (A)
WNT, fusion of the name of the Drosophila segment polarity gene *wingless* and the name of the vertebrate homolog: *integrated or int-1*
XBP1: x-box–binding protein 1
YAP: yes-associated protein
ZEB1: zinc finger E-box binding homeobox 1
ZEB2: zinc finger E-box binding homeobox 2.
